# Resonant vortex-core reversal in magnetic nano-spheres as robust mechanism of efficient energy absorption and emission

**DOI:** 10.1038/srep31513

**Published:** 2016-08-17

**Authors:** Sang-Koog Kim, Myoung-Woo Yoo, Jehyun Lee, Jae-Hyeok Lee, Min-Kwan Kim

**Affiliations:** 1 National Creative Research Initiative Center for Spin Dynamics and Spin-Wave Devices, Nanospinics Laboratory, Research Institute of Advanced Materials, Department of Materials Science and Engineering, Seoul National University, Seoul 151-744, South Korea

## Abstract

We report on novel vortex-core reversal dynamics in nano-spheres of single-vortex spin configuration as revealed by micromagnetic simulations combined with analytical derivations. When the frequency of an AC magnetic field is tuned to the frequency of the vortex-core precession around the direction of a given static field, oscillatory vortex-core reversals occur, and additionally, the frequency is found to change with both the strength of the applied AC field and the particle size. Such resonant vortex-core reversals in nano-spheres may provide a new and efficient means of energy absorption by, and emission from, magnetic nanoparticles, which system can be effectively implemented in bio-applications such as magnetic hyperthermia.

Non-uniform spin configurations in magnetic nano-elements exhibit contrasting magnetization reversal dynamics. For instance, the magnetic vortex, which consists of a uniformly magnetized core in a local central region and curling spin configuration around it in planar thin-film nano-dots^1^,^2^, show, through the resonant dynamic effect, novel vortex-core gyration and switching dynamics with low-power consumption. Such novel dynamics as the mechanism^3^,^4^,^5^, criterion^6^,^7^,^8^,^9^,^10^,^11^, and physical origin^4^,^8^,^11^ of the core reversals are well established in case of planar thin-film disks. As for soft-magnetic nano-spheres, our previous papers^12^,^13^ reported that they bear specific spiral magnetizations around a uniformly magnetized core region, the so-called vortex core, in cases where the particle diameter is larger than single-domain size but smaller than multiple-domain size. In our earlier work^12^, we demonstrated successful fabrication of spherical Permalloy (Py) nanoparticles and conformed vortex-state magnetization configurations using the electron holography measurement technique. As shown in ref. 13, the vortex core in nano-spheres is found to exhibit a unique precession motion around the direction of an externally applied static field, and, further, the precession frequency is determined by the sphere size as well as the static field strength. The core precession thus can be excited with low-power consumption when the frequency of an externally applied AC magnetic field is tuned to the eigenfrequency of the core-precession mode, though the mechanism is completely different from that of vortex-core gyration in planar thin-film dots.

In the present study, we explored vortex-core reversals resonantly driven by AC magnetic fields with the assistance of excited vortex-core precession in soft magnetic nano-spheres. This vortex-core reversal is completely different from typical microwave-assisted magnetization switching in single-domain nanoparticles and vortex-core-gyration-assisted core reversals in planar thin-film dots. The relevant dynamics serve as a robust mechanism of efficient energy transfer into and from vortex-state magnetic particles: application of sufficiently weak AC magnetic fields allows for resonant energy absorption from external magnetic fields to vortex-state nano-spheres. Although the vortex-core precession in nano-spheres and its frequency dependence on particle size and the strength of the DC field have already been reported (see ref. 13), the vortex-core reversal and its underlying physics have as yet remained (until the present work) unexplored. The simulation and analytical numerical calculations in the present work revealed that the core-reversal frequency changes significantly with the AC field strength for a given particle diameter. The volume or mass-specific energy absorption rate of the magnetic nano-spheres showed good agreement with the simulation results. Such an efficient means of high-power-rate energy absorption and subsequent emission can be implemented in possible future bio-diagnostic and magnetic-hyperthermia-treatment applications.

## Results

### Resonant vortex-core reversal

**Figure 1:**
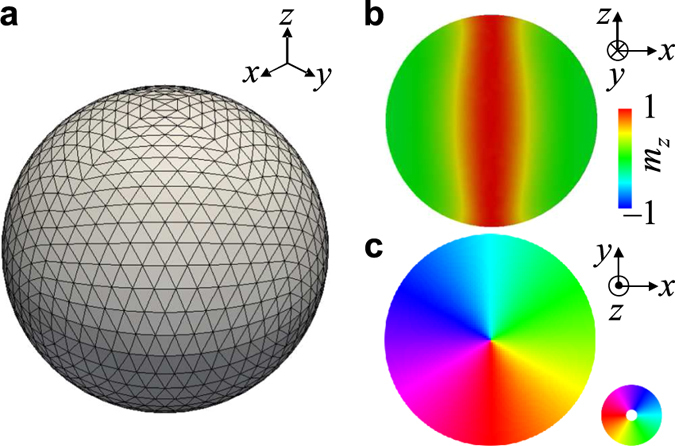
Sphere model and its ground-state magnetization configuration. (**a**) Finite-element Py sphere of diameter 2*R* = 80 nm. (**b**,**c**) represent the ground-state magnetization configuration viewed in the *x*-*z* plane at *y* = 0 nm and the *x*-*y* plane at *z* = 0 nm, respectively. The colors in (**b**,**c**) correspond to the *z* components of the local magnetizations (*m*_*z*_ = *M*_*z*_/*M*_s_), as indicted by the color bar, and to the in-plane components of the local magnetizations, as indicated by the color wheel.

**Figure 2:**
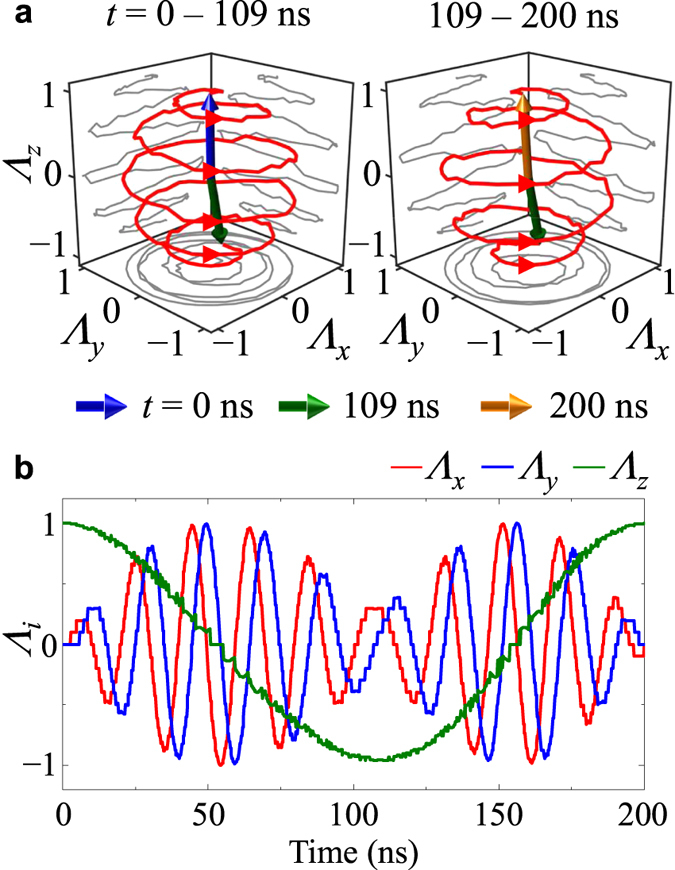
Temporal evolution of vortex-core orientation during resonant core precession and reversal. (**a**) Trajectories (red color) of vortex-core orientation **Λ** = (*Λ*_*x*_, *Λ*_*y*_, *Λ*_*z*_), which motion is resonantly excited by a circular rotating field, 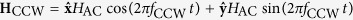 with *H*_AC_ = 10 Oe and *f*_CCW_ = 51 MHz, while applying a static field along the *z* axis (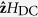, *H*_DC_ = 100 Oe). The left and right panels correspond to the core motions during *t* = 0‒109 ns and *t* = 109‒200 ns, respectively. The blue, green, and orange arrows represent the core orientation at *t* = 0, 109, and 200 ns, respectively. (**b**) Temporal variation of *x*, *y*, *z* components of core orientations, *Λ*_*x*_, *Λ*_*y*_, and *Λ*_*z*_.

Figure 1 shows the ground state of a permalloy (Py) nano-sphere of 2*R* = 80 nm diameter, as obtained by relaxation from the saturated magnetizations in the +*z*-direction. The spin microstructure indicates a rather uniformly magnetized vortex core with spiral magnetizations around the core. To resonantly excite the core precession, we applied a counter-clockwise (CCW) circular-rotating field, owing to the fact that in the sphere, one of the intrinsic dynamic modes is the CCW core precession about a DC magnetic field applied in the +*z*-direction. The CCW circular-rotating field used is expressed mathematically as 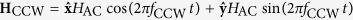, where *f*_CCW_ is the frequency of the rotating field with a strength of *H*_AC_ = 10 Oe and a static magnetic field 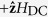 (here *H*_DC_ = 100 Oe). When we applied *f*_CCW_ = 51 MHz, which equaled the eigenfrequency of the core precession (See Methods), the core started to show precession, and then reversed its magnetization orientation at a certain time with respect to the direction of 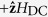 (see Supplementary Movie). Figure 2a plots, in the 3D perspective, the trajectory of the core motion as represented by the unit vector **Λ** of the core orientation. The temporal evolutions of the *x*-, *y*-, *z*- components of **Λ** are shown in Fig. 2b. In detail, upon the application of the CCW rotation field, the core, of initial orientation **Λ** = (0, 0, 1) at *t* = 0 ns, begins its precession about the +*z*-direction in CCW rotational sense. The angle between **Λ** and 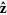 increases with time until, at *t* = 109 ns, the core orientation flips to **Λ** = (0, 0, −1). This core-reversal behavior discovery is the first for vortex-state nano-spheres. With continuous application of the CCW rotating field, the reversed core returns, at *t* = 200 ns, to the initial **Λ** = (0, 0, 1) orientation via core precession of the same CCW rotation sense. This reversal occurs repeatedly in a periodic manner. From the FFTs of the temporal variations of the *x*-, *y*-, *z*-components of **Λ** shown in Fig. 2b, we obtained the frequencies of the core precession and reversal to be *f*_prec_ ~ 51 MHz and *f*_rev_ ~ 5 MHz respectively, for the given diameter of 80 nm and *H*_DC_ = 100 Oe. We stress here that the mechanism of periodic core reversal via resonantly excited core precession differs completely not only from that of microwave-assisted magnetization switching in single-domain magnetic elements^14^,^15^,^16^,^17^,^18^,^19^,^20^ but also from that of vortex-core reversals driven by oscillating^5^,^6^,^8^,^21^ or pulse^22^,^23^ fields/currents in planar thin-film dots.

**Figure 3:**
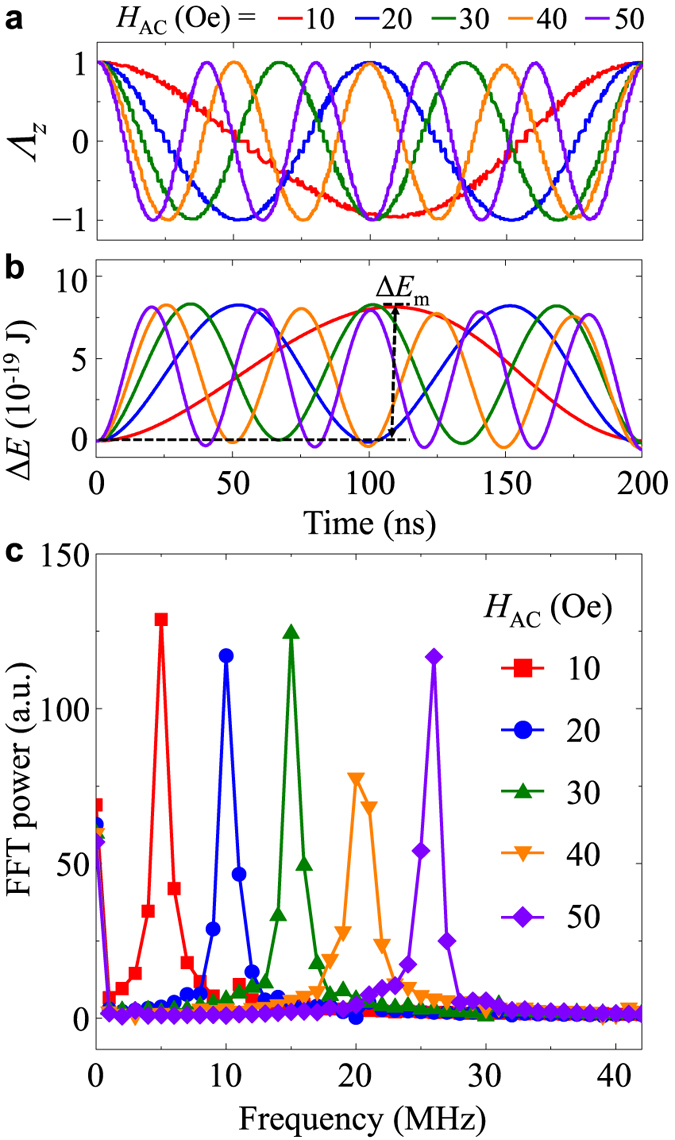
Core-reversal oscillation versus ac field strength. (**a**) Temporal evolution of *Λ*_*z*_ for different field strengths, *H*_AC_ = 10, 20, 30, 40, and 50 Oe. (**b**) Total energy variation (Δ*E* = *E*(*t*) − *E*(0)) during resonant core reversal in Py nano-spheres of 2*R* = 80 nm with *H*_DC_ = 100 Oe. Δ*E*_m_ denotes the maximum energy increment for each *H*_AC_. (**c**) FFT power spectrum in frequency domain for different *H*_AC_ values, as obtained from *Λ*_*z*_ oscillations in (**b**) within *t* = 0 − 1 μs period.

**Figure 4:**
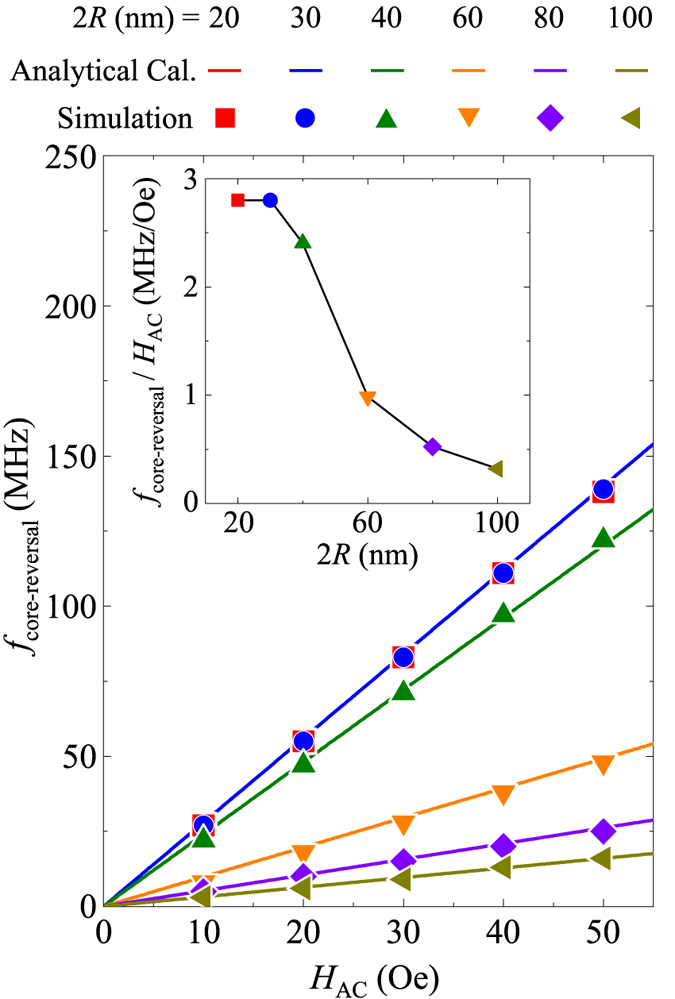
Core-reversal frequency as function of *H*_AC_ for different 2*R* values. The symbols denote the micromagnetic simulation results, while the solid lines correspond to the numerical calculations using 2*πf*_rev_ = *γ*〈*m*_Λ_〉*H*_AC_. The inset shows the corresponding slopes of the *f*_rev_ versus *H*_AC_ linear curves for the different 2*R* values.

Next, we examined the variation of *f*_rev_ with *H*_AC_. Figure 3a plots the temporal oscillations of *Λ*_*z*_ for different *H*_AC_ values, and Fig. 3b the total energy variation, during core precession and reversal. The FFTs of the *Λ*_*z*_ oscillations result in characteristic resonant peaks in FFT power versus *f*_AC_ according to *H*_AC_ (see Fig. 3c). Those peaks are sufficiently distinguishable with varying *H*_AC_ in steps of 10 Oe, indicating that the core reversal’s frequency varies and is controllable with the strength of *H*_AC_, not with the static field strength *H*_DC_. This fact is key to the control of *f*_rev_ simply with the strength of *H*_AC_ for a given nano-sphere. With respect to one of our earlier studies, we reported that *f*_prec_ varies with the sphere diameter, 2*R*. Thus, in the present investigation, we also examined, in further simulations, the variation of *f*_rev_ with 2*R*. Figure 4 shows the linear relations between *f*_rev_ and *H*_AC_ but different slopes for different 2*R* values. For each diameter, we used the *f*_CCW_ value that equals the *f*_prec_ for the given 2*R*. The slope (*f*_rev_/*H*_AC_) is plotted as a function of 2*R* in the inset of Fig. 4. As can be seen, as 2*R* increases, the slope markedly decreases. For example, for single-domain states (2*R* = 20 and 30 nm), the slope is about 2.8 MHz/Oe but for 2*R* = 60 nm, it decreases to 0.985 MHz/Oe. This value is about 65% smaller than that for single-domain spheres. It is noteworthy too, that for single-domain spheres, *f*_rev_/*H*_AC_ does not change with 2*R*, while for vortex-state spheres, it varies significantly with 2*R*. From a technological point of view, it is advantageous that the resonant core reversal is very specific to the particle size for a given strength of *H*_AC_. This means, crucially, that size-specific particle excitations, including core precession and reversal, are possible.

### Analytical derivation

To understand the underlying physics of the dependences of *f*_rev_ on both *H*_AC_ and 2*R*, as revealed by the micromagnetic simulations, we derived the analytical form of *f*_rev_ as a function of both 2*R* and *H*_AC_. By assuming a rigid vortex model and negligible Gilbert damping, we obtained the governing equation of the core motion in a vortex-state nano-sphere, 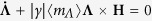, where **H** is the external magnetic field and 〈*m*_Λ_〉 is the average magnetization component over the sphere volume in the vortex-core orientation (see Methods for details). As in the micromagnetic simulations, we set **H** = 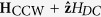 with 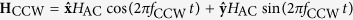. By setting *f*_CCW_ = *f*_prec_ and the initial core orientation as **Λ** = (0, 0, 1) at *t* = 0, we obtained the relations


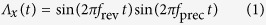



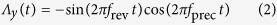



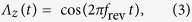


where 2*πf*_rev_ = *γ*〈*m*_Λ_〉*H*_AC_ and 2*πf*_prec_ = *γ*〈*m*_Λ_〉*H*_DC_. As reported in ref. 13, the value of 〈*m*_Λ_〉 varies markedly with 2*R* in vortex-state spheres (〈*m*_Λ_〉  = 1 for single-domain spheres). In the present results, it was quite noticeable that the core reversal’s frequency is a function of both 〈*m*_Λ_〉 and *H*_AC_. The numerical calculation (i.e., analytical expression) of 2*πf*_rev_ = *γ*〈*m*_Λ_〉*H*_AC_ for different 2*R* values agreed well with the simulation results, as represented by the lines and symbols respectively, in Fig. 4. In the numerical calculation, we used 〈*m*_Λ_〉 = 73.6(*l*_ex_/2*R*)^2.20^ with *l*_ex_ the exchange length (*l*_ex_ ~ 5.3 nm for Py), as reported in ref. 13. For the given material Py, *f*_rev_ is therefore a function of both *H*_AC_ and 2*R* for the vortex-state spheres, but only of *H*_AC_ for the single-domain spheres (i.e., 〈*m*_Λ_〉 = 1), independently of the particle size, as shown in the inset of Fig. 4.

### Robust mechanism of efficient energy absorption and emission

Controllable nano-sphere vortex-core reversals driven by oscillation or pulse fields in vortex-state soft magnetic nanoparticles can be used as a robust mechanism of resonant energy absorption and emission. Via the novel dynamic effects of the observed resonant core precession and reversal, external magnetic field energy can be transferred efficiently to nano-spheres. Furthermore, the energy absorbed into nanoparticles can be emitted to proximate environments in the form of heat through magnetization dissipation^24^,^25^ (or via electromagnetic waves, though the energy is very weak). Such energy-exchange processes are efficiently achieved, via the resonant excitation of vortex-core precession and reversal, by tuning the frequency of the AC field, whether circular rotating or linear, to the *f*_prec_ value for a given material, diameter, and *H*_DC_.

**Figure 5:**
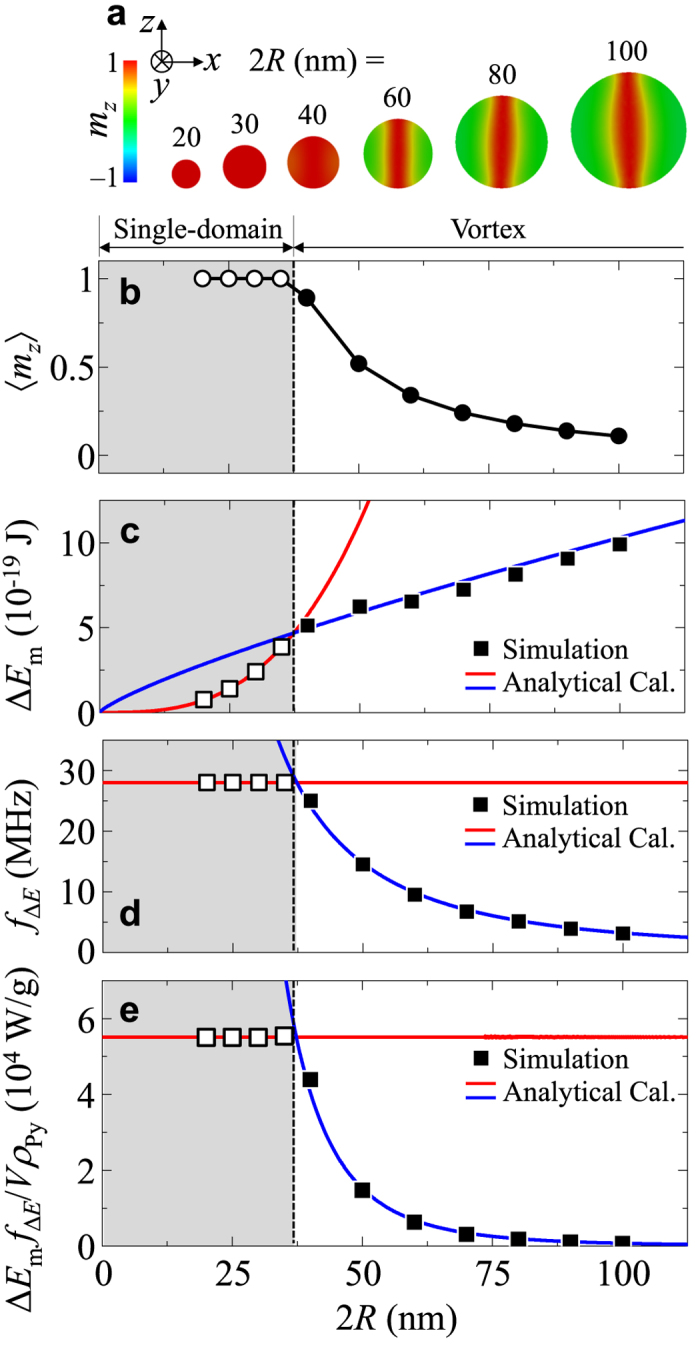
Energy absorption rate by resonant core reversal as function of 2*R*. (**a**) Ground-state magnetization configurations in Py nano-spheres of given diameters, as viewed in *x*-*z* plane at *y* = 0 nm. The core orientations are indicated by the *m*_*z*_ color bar. (**b**–**e**) represent 〈*m*_z_〉, Δ*E*_m_, *f*_Δ*E*_, and Δ*E*_m_ *f*_Δ*E*_/*Vρ*_Py_ as functions of 2*R*, as obtained from the micromagnetic simulation results (square symbols) and the numerical calculations (solid lines) of the analytical forms expressed in the text. The uniform single-domain state (gray region) and vortex state (white region) are distinguished by the vertical dotted line at 2*R* = 37.3 nm.

In order to quantitatively determine the amount of the energy exchange and its transfer rate (i.e., power), we calculated the energy variation Δ*E* as a function of time for different *H*_AC_ values, as shown in Fig. 3b. The periodicities of the Δ*E* oscillations exactly equaled those of the *Λ*_*z*_ oscillations; thus *f*_Δ*E*_ = *f*_rev_. The maximum energy increment Δ*E*_m_ is constant with *H*_AC_. The 2*R* dependence of Δ*E*_m_ differs from the single domain to the vortex-state spheres, as indicated in Fig. 5c by the open and closed symbols, respectively. Additionally, for the single-domain spheres (here 2*R* ≤ 37.3 nm), the frequency of Δ*E* oscillation (the top panel of Fig. 5d) is a constant value of 280 MHz, whereas for the vortex-state nano-spheres, it markedly varies with 2*R*. The energy absorption rate per unit mass (i.e., the power per mass), defined as Δ*E*_m_ *f*_Δ*E*_/*Vρ*_Py_, was calculated versus 2*R* from the simulation data using *ρ*_Py_ = 8.74 g/cm^3^ for Py, as shown in Fig. 5e. The energy absorption rate (EAR) versus 2*R* for the vortex-state particles differs from a constant value of 5.5 × 10^4^ W/g for the single-domain-state particles. The EAR is similar to the specific absorption rate (SAR) representative of heating efficiency in magnetic hyperthermia^26^,^27^.

To obtain further insight into the 2*R*-dependent EAR, we analytically derived the EAR as defined by Δ*E*_m_ *f*_Δ*E*_/*Vρ*_Py_. As explained earlier, Δ*E* oscillations originate from the Zeeman energy variation due to the reversal of the core orientation with respect to the +z direction of the applied *H*_DC_. Thus, Δ*E*_m_ can be rewritten in the form 2*VM*_s_〈*m*_Λ_〉*H*_DC_^13^ along with *f*_Δ*E*_ = (*γ*/2*π*)〈*m*_Λ_〉*H*_AC_. By applying 〈*m*_Λ_〉 = 1 for the single-domain and 〈*m*_Λ_〉 = 73.6(*l*_ex_/2*R*)^2.2^ for vortex-state particles, we obtained the analytic forms of 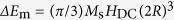 and 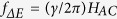 for single-domain particles and of 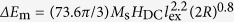 and 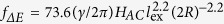 for vortex-state particles. From these analytical expressions, we finally obtained 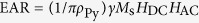 for single-domain particles and 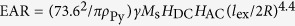 for vortex-state particles. As shown in Fig. 5c–e, these analytic forms (the corresponding lines) were found to be in excellent agreement with the simulation results (the square symbols) for Py nano-spheres of different 2*R*.

As shown in Fig. 5e, the EAR per mass for single-domain Py particles is about 5.5 × 10^4^ W/g, which is one or two orders of magnitude larger than typical SAR value (~10^2^–10^3^ W/g) for magnetic hyperthermia^28^. The EAR for vortex-state particles varies with 2*R*, as indicative of particle-size specificity. Furthermore, for a given size of vortex-state nano-sphere, the specific *H*_AC_ and *H*_DC_ and values also determine *f*_Δ*E*_ = *f*_rev_ and Δ*E*_m_, respectively, thus characterizing specific EAR values. This factor, crucially, provides for vortex-state-nanoparticle-based EAR controllability not only by nanoparticle size but also, and simply, by tunable parameters (e.g., field strength and frequency) of externally applied AC and DC magnetic fields.

## Discussion

We studied soft magnetic nano-spheres’ vortex-core reversals assisted by the resonantly excited vortex-core precession. We found a novel dynamic behavior, which is the high dependence of the periodic core-reversal frequency on the strength of external AC magnetic fields as well as particle size. Although the core precession and its frequency dependence on the DC magnetic field and the particle size were explored in ref. 13, the aforementioned core reversal and its underlying physics have yet to be unveiled; thus, in the present work, we derived analytic forms of the core-reversal frequency as a function of the sphere diameter and AC field strength.

We suggest that these vortex-core-reversal dynamics’ possible application to magnetic hyperthermia can provide benefits such as particle-size specificity and power efficiency. For example, magnetic nanoparticles can be guided to specific areas such as tumors, where the particles can efficiently absorb energy in the form of an AC magnetic field when its frequency corresponds to the resonant core-reversal frequency. Chemical functionalizations of bio-compatible materials such as magnetic iron oxides^29^ can make this unique mechanism more technically available to bio-applications. In fact, because the magnetic EAR we calculated for vortex-core reversals of soft magnetic nano-spheres is of sufficiently high value and very specific to a given particle size as well as a given AC field strength, the vortex-core reversal dynamics might be a promising candidate for robust hyperthermia therapy of great power efficiency and/or entailing only the minimal intake of nanoparticles.

Several reports regarding the application of the magnetic vortex to bio-applications have been published. Kim *et al.*^30^ experimentally demonstrated a protocol for *in vitro* cancer therapy with bio-functionalized magnetic vortex-state discs. The main mechanism is the transmission of a mechanical force induced by application of oscillating magnetic fields of a few tens of Hz to compromise membrane integrity and initiate magnetic-vortex-mediated cancer-cell destruction. Another notable implementation of magnetic hyperthermia utilizing the magnetic vortex was achieved by Liu *et al.,*^31^ who reported an enhanced therapeutic efficacy for hyperthermal tumor treatment with ferromagnetic vortex-domain nano-rings. However, from the standpoint of micro-magnetism, the two preceding studies above mentioned are in the regime of static responses that are usually controlled by a static magnetic field or an AC field of significantly low frequency range of 10 Hz–400 kHz. Our method, which differs completely from those two (notwithstanding their correspondent use of vortex states in magnetic nanoelements), utilizes the robust resonant dynamic characteristics of vortex-core reversals newly discovered in the present work. This work thus provides deeper insight into the fundamentals of vortex-core dynamics in soft-magnetic nano-spheres, and suggests, further, a technical new route to the achievement of efficient modes of magnetic-hyperthermia-applicable energy absorption and emission.

## Methods

### Micromagnetic simulation

We performed finite-element micromagnetic simulations of the magnetization dynamics of Py nano-spheres of varying diameter, 2*R* = 20–100 nm. For the numerical calculations of motions of the local magnetizations, we used the FEMME code (version 5.0.9)^32^, which utilizes the LLG equation. To prevent surface-irregularity-incurred numerical sphere-model errors, we discretized the surface of the nanosphere into triangles of roughly equal area using Hierarchical Triangular Mesh^33^ as well as the inner volume into tetrahedral meshes (mesh size ≤ 5 nm) (see Fig. 1a). The magnetic parameters for the soft ferromagnetic Py material were as follows: saturation magnetization *M*_s_ = 860 emu/cm^3^, exchange stiffness *A* = 1.3 × 10^−6^ erg/cm, Gilbert damping constant *α* = 0.01, gyromagnetic ratio *γ* = 2*π* × 2.8 MHz/Oe, and zero magneto crystalline anisotropy.

### Determination of eigenfrequency of vortex-core precession

To estimate the eigenfrequency of the core precession in a nano-sphere of specific diameter, we applied a static magnetic field of 100 Oe in the +*x* direction for 1 μs to the initial ground vortex state of the core aligned in the +*z*-direction. Then, we extracted the value of 〈*m*_*z*_〉, defined as the average of *m*_*z*_ (= *M*_*z*_/*M*_s_) over the sphere volume during the precession. The core-precession frequency was determined from the Fast Fourier Transformation (FFT) of the temporal variation of 〈*m*_z_〉.

### Analytical derivation

The core motion of the rigid vortex in a soft-magnetic nano-sphere can be expressed as^13^


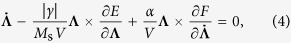


where **Λ** is the unit vector of the core orientation, *E* the total magnetic energy, *F* the dissipative term, and *V* the volume of a sphere. The first, second and third terms correspond to the core motion, the potential energy and damping terms, respectively. The energy *E* is given as 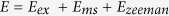, with *E*_ex_ the exchange energy, *E*_ms_ the magnetostatic energy, and *E*_Zeeman_ the Zeeman energy. In order to derive the frequency of the periodic core reversal, the damping term can be neglected so that Eq. (4) simply becomes 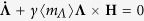 with 
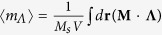
.

## Additional Information

**How to cite this article**: Kim, S.-K. *et al.* Resonant vortex-core reversal in magnetic nano-spheres as robust mechanism of efficient energy absorption and emission. *Sci. Rep.* **6**, 31513; doi: 10.1038/srep31513 (2016).

## Supplementary Material

Supplementary Movie

Supplementary Information
