# Supplementary material for: Resonant vortex-core reversal in magnetic nano-spheres as robust mechanism of efficient energy absorption and emission
Source: Sci Rep. 2016 Aug 17;6:31513. doi: 10.1038/srep31513 (PMC4987621; doi:10.1038/srep31513)
Supplement: Supplementary Information [file srep31513-s2.pdf]

Resonant vortex-core reversal in magnetic nano-spheres as robust mechanism of efficient energy absorption and emission

Sang-Koog Kim[a)], Myoung-Woo Yoo, Jehyun Lee[+], Jae-Hyeok Lee, and Min-Kwan Kim

National Creative Research Initiative Center for Spin Dynamics and Spin-Wave Devices, Nanospinics Laboratory, Research Institute of Advanced Materials, Department of Materials Science and Engineering, Seoul National University, Seoul 151-744, South Korea

[a)] Correspondence and requests for materials should be addressed to S.-K.K. (email: sangkoog@snu.ac.kr).

[+] Present address: Center of Semiconductor Research & Development, Gyeonggi-do 445-701, South Korea

Supplementary Movie

Supplementary movie presents a dynamic motion of vortex-core reversal in the model sphere of $2R$ = 80 nm, as driven by H_{CCW} = 10 Oe at f_{CCW} = 51 MHz under a static field of H_{DC} = 100 Oe in the +z direction. The red-, orange- and blue-color arrows represent the orientations of the vortex core, the static magnetic field, and the counter-clockwise circular-rotating field, respectively.
